# The Emerging Role of Autophagy-Associated lncRNAs in the Pathogenesis of Neurodegenerative Diseases

**DOI:** 10.3390/ijms24119686

**Published:** 2023-06-02

**Authors:** Yapei Jiang, Naihan Xu

**Affiliations:** 1 State Key Laboratory of Chemical Oncogenomics, Tsinghua Shenzhen International Graduate School, Tsinghua University, Shenzhen 518055, China; jiang_yapei@163.com; 2 Open FIESTA Center, Tsinghua Shenzhen International Graduate School, Tsinghua University, Shenzhen 518055, China; 3 Institute of Biopharmaceutical and Health Engineering, Tsinghua Shenzhen International Graduate School, Tsinghua University, Shenzhen 518055, China

**Keywords:** lncRNA, autophagy, neurodegenerative disease, Alzheimer’s disease, Parkinson’s disease

## Abstract

Neurodegenerative diseases (NDDs) have become a significant global public health problem and a major societal burden. The World Health Organization predicts that NDDs will overtake cancer as the second most common cause of human mortality within 20 years. Thus, it is urgently important to identify pathogenic and diagnostic molecular markers related to neurodegenerative processes. Autophagy is a powerful process for removing aggregate-prone proteins in neurons; defects in autophagy are often associated with the pathogenesis of NDDs. Long non-coding RNAs (lncRNAs) have been suggested as key regulators in neurodevelopment; aberrant regulation of lncRNAs contributes to neurological disorders. In this review, we summarize the recent progress in the study of lncRNAs and autophagy in the context of neurodegenerative disorders, especially Alzheimer’s disease (AD) and Parkinson’s disease (PD). The information presented here should provide guidance for future in-depth investigations of neurodegenerative processes and related diagnostic molecular markers and treatment targets.

## 1. Introduction

Long non-coding RNAs (LncRNAs) are transcribed by RNA polymerase II and structurally resemble mRNA molecules, with sizes greater than 200 nt in length [1]. To date, over 50,000 human lncRNAs have been identified that span 68% of the human genome. Most lncRNAs lack open reading frames (ORFs) and thus do not generally encode proteins (except for a small number of functional peptides) [2,3]. LncRNA sequences are poorly conserved among different species [4,5]. The vast majority of lncRNAs are expressed at lower levels in comparison with protein-coding mRNAs, due to relatively lower synthesis, processing and stability than protein-coding mRNAs [6]. It is well known that lncRNAs regulate gene expression with diverse molecular mechanisms, including intracellular signaling, decoy, guidance and scaffold functions [7]. LncRNAs are pivotal regulators of gene expression and genome structure. By interacting with DNA, RNA and proteins, lncRNAs are involved in chromatin remodeling, transcription and post-transcriptional regulation, thus controlling various biological processes such as proliferation, differentiation, apoptosis, autophagy, etc. [8,9]. Due to their diverse cellular functions, lncRNAs are implicated in the pathological processes of numerous human diseases, including neurodegenerative diseases (NDDs) [10]. Results of numerous studies reported that lncRNAs play a direct role in synaptic plasticity, memory gene regulation and cognitive function and have an integral function in normal brain development and the maintenance of brain function. Thus, lncRNAs emerge as potential diagnostic and therapeutic targets of NDDs [11].

Autophagy, a highly conserved cellular self-digestive process that occurs in eukaryotic cells, is essential for maintaining homeostasis and supporting self-repair. Autophagy plays a key role in regulating various cellular physiological processes; autophagy defects contribute to the pathogenesis of cancer, metabolic disorders, diabetes, NDDs, cardiovascular diseases, immune diseases, etc. [12,13]. Neuronal cells rely heavily on autophagy to maintain homeostasis [14]. Autophagy plays key roles in degrading aggregate-prone proteins. Improper intracellular and extracellular accumulations of mutated and misfolded neuronal proteins are the most obvious hallmarks of NDDs, such as Aβ and C-terminal fragments (CTFs) of the amyloid precursor (APP) in Alzheimer’s disease (AD), mutant alpha-synuclein in Parkinson’s disease (PD), mutant TAR DNA-binding protein 43 (TDP-43) in amyotrophic lateral sclerosis (ALS), mutant huntingtin protein (mHTT) with expanded polyglutamine in Huntington’s disease (HD), etc. [15,16]. Most aggregated pathogenic mutant proteins associated with NDDs are mainly targeted by the autophagy–lysosome pathway [17]. Dysregulated autophagy causes neuronal cell degeneration and apoptosis, leading to the development of NDDs [18,19]. Therefore, targeting autophagy is becoming a promising strategy for these diseases.

**Figure 1:**
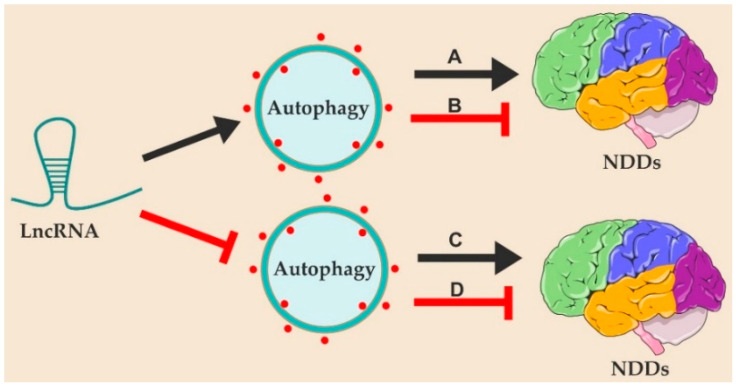
Four forms of functional cross-regulation between lncRNAs and autophagy in NDDs: (A) LncRNAs promote NDDs by activating autophagy. (B) LncRNAs inhibit NDDs by activating autophagy. (C) LncRNAs promote NDDs by suppressing autophagy. (D) LncRNAs inhibit NDDs by suppressing autophagy.

There has been accumulating evidence that lncRNAs are critical regulators of autophagy. LncRNAs generally function as competitive endogenous RNAs (ceRNAs) to regulate the expression of autophagy-related genes. In this review, we summarize recent advances in understanding the relationship between lncRNA and autophagy regulation in the context of neurodegenerative disorders. As autophagy is a double-edged sword, excessive or insufficient autophagy in neuronal cells can lead to homeostatic imbalance and neurodegeneration [20]. LncRNAs either promote or inhibit the progression of NDDs by activating or suppressing autophagic activity in neuronal cells under different circumstances (Figure 1). We also briefly outline some of the latest statuses of treatments and the potential challenges of lncRNA in therapeutic interventions.

## 2. LncRNAs Regulate the Pathogenesis of Neurodegenerative Diseases by Modulating Autophagy

### 2.1. LncRNAs in the Pathogenesis of Neurodegenerative Diseases

It has been reported that nearly 40% of lncRNAs are specifically expressed in the brain; brain lncRNAs are highly conserved and show stronger temporal and spatial specificity than protein-coding genes [21]. A growing number of studies have conducted in-depth comparisons of lncRNA expression profiles between NDD patients and normal individuals. Zhou et al. identified 24 upregulated and 84 downregulated lncRNAs in AD patients [22]. Ni et al. identified 87 deregulated lncRNAs within nigrostriatal tissues of PD patients, of which 42 exhibited significantly elevated expression, and 45 downregulated relative to healthy controls [23]. Johnson R. reported seven lncRNAs that exhibited dysregulated expression in HD patients [24]. Gagliardi S. et al. revealed 293 differentially expressed lncRNAs in sporadic ALS patients, while only 21 lncRNAs were detected in familial ALS patients [25]. All above-mentioned lncRNAs are identified based on differential expression profiles; additional experimental studies are required to confirm the function and molecular mechanisms of these lncRNAs in the pathogenesis of NDDs. At present, results obtained from published papers indicate that approximately 31 lncRNAs have been extensively studied to play significant roles in AD, and nearly 34 lncRNAs are experimentally identified to regulate pathogenesis in PD. However, only four lncRNAs are described to play critical roles in the development of HD; very few lncRNAs have been reported in ALS (Table 1). Many of the lncRNAs mentioned above have the potential to be biomarkers for the diagnosis of neurodegenerative disorders.

NEAT1 (nuclear paraspeckle assembly transcript 1) and MALAT1 (metastasis-associated lung adenocarcinoma transcript-1) are the most frequently studied lncRNAs in NDDs. Multiple lines of evidence indicate NEAT1 was significantly upregulated in AD, PD, HD and ALS. NEAT1 has been shown to modulate the expression of Aβ, α-synuclein, mHTT and TDP-43 via sponging microRNA, transcriptional and translational regulation and protein modification. The roles of NEAT1 in disease progression are diverse. NEAT1 can promote or delay the progressive loss of neurons, which depends on multiple downstream targets of NEAT1 [113]. MALAT1 plays important roles in normal brain development; the aberrant expression of MALAT1 was observed in central nervous system (CNS) disorders, including AD and PD. Lower levels of MALAT1 were detected in the cerebrospinal fluid of AD patients [114]. MALAT1 was significantly upregulated in an MPTP-induced PD mice model and PD patients and can be considered as a diagnostic and prognostic biomarker. MALAT1 dysregulation is closely associated with α-synuclein aggregation and Lewy body formation [115].

### 2.2. LncRNAs Regulate Alzheimer’s Disease by Modulating Autophagy

Alzheimer’s disease, one of the most common chronic NDDs afflicting the elderly, damages neurons within the brain and leads to cognitive degeneration [116]. According to Alzheimer’s Disease International (ADI), more than 50 million people worldwide were suffering from AD in 2020, with the number of Alzheimer’s patients expected to exceed 100 million by 2050 [117]. The pathological process underlying AD is unknown but is thought to involve a highly complex mechanism. Currently, the leading hypothesis on the pathophysiology of AD is the beta-amyloid protein (Aβ) and misfolded microtubule-related tau protein molecules that accumulate in the neurons of AD patients. These abnormal proteins cannot be cleared effectively and ultimately damage mitochondria and other cellular components, resulting in the disruption of neuronal cell physiological function, cell death and brain damage [118,119].

Results from numerous studies suggest that lncRNAs regulate AD pathogenesis by modulating the autophagy pathway. Autophagy has been broadly recognized as a double-edged sword in Alzheimer’s disease. Some studies have suggested that lncRNA can exacerbate AD progression by inhibiting autophagy. For instance, lnc17A was significantly upregulated in an AD cell model; the knockdown of lnc17A expression reduced Aβ1-42 accumulation by activating autophagy activity [57]. NEAT1 was significantly upregulated in an AD mouse model and promoted the accumulation of amyloid-β. NEAT1 can directly bind to both the E3 ubiquitin ligase NEDD4L and the mitophagy regulator PINK1 (PTEN-induced kinase 1) to promote proteasomal degradation of PINK1, resulting in the disruption of PINK1-dependent mitophagy and amyloid accumulation [120]. LncRNA MIR600HG expression was elevated during aging in AD transgenic mice; MIR600HG interacted with NEDD4L to promote PINK1 ubiquitination and degradation, thus inhibiting PINK1-mediated mitophagy and preventing clearance of amyloid-β. The knockdown of MIR600HG markedly ameliorated the cognitive impairment in AD mice [58]. Interestingly, Jia et al. reported that moxibustion can inhibit the expression of lncRNA SIX3OS1 in AD mice; silencing SIX3OS1 promoted autophagy and accelerated Aβ1-42 clearance by inactivating PI3K/AKT/mTOR signaling [62].

LncRNAs can contribute to the development of Alzheimer’s disease by activating autophagy. Results of several studies have demonstrated that the inhibition of autophagy can reduce the deterioration of hippocampal neurons, thus alleviating cognitive decline [121]. LncRNA RMRP (the RNA component of mitochondrial RNA-processing endoribonuclease) was highly enriched in sera collected from hippocampal tissues of AD patients and AD mice. Knockdown RMRP expression alleviated neuronal cell apoptosis by suppressing autophagy. RMRP could act as a molecular sponge of miR-3142 to elevate the expression of TRIB3 (Tribbles pseudokinase 3). TRIB3 was also abnormally upregulated in AD; the overexpression of TRIB3 reversed the effect of RMPP silencing on Aβ1-42-induced neuronal cell apoptosis and autophagy [59]. LncRNA BACE1-AS was upregulated in the serum of AD patients, brain tissues of AD mice and an Aβ1–42-treated SH–SY5Y cell model for AD [122]. BACE1-AS upregulated the expression of ATG5 by sponging miR-214-3p. The knockdown of BACE1-AS alleviated Aβ1–42-induced neuronal cell injury by repressing autophagy through the miR-214-35/ATG5 axis in AD [60].

**Figure 2:**
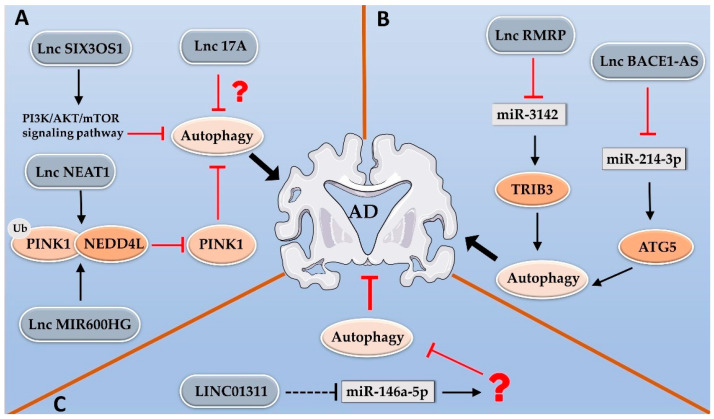
LncRNAs contribute to the pathogenesis of AD by modulating the autophagy pathway. Three different forms of functional cross-regulation between lncRNAs and autophagy are observed in AD: (**A**) LncRNA 17A, NEAT1, MIR600HG and SIX3OS1 accelerate the progression of AD by inhibiting autophagy. Both NEAT1 and MIR600HG bind to NEDD4L to promote the proteasomal degradation of PINK1 and inhibit mitophagy; SIX3OS1 inhibits autophagy by activating the PI3K/AKT/mTOR pathway. The distinct role of lnc-17A in autophagy inhibition is unknown. (**B**) LncRNA RMRP and BACE1-AS1 promote the development of AD through sponging miR-3142 or miR-214-3p to elevate the expression of TRIB3 or ATG5 to activate autophagy. (**C**) LINC01311 suppresses AD progression through sponging miR-146a-5p and inhibiting autophagy. The distinct function of miR-146-5p in neuronal cell autophagy is unknown.

On the other hand, lncRNAs could also suppress the progression of Alzheimer’s disease by inhibiting autophagy. LINC01311 was downregulated in an Aβ1–42-treated SH–SY5Y cell model for AD. The enforced expression of LINC01311 protected cells from Aβ1–42-induced autophagy and neuronal cell apoptosis. Mechanistically, LINC01311 acts as a ceRNA of miR-146a-5p, and the overexpression of miR-146a-5p reversed the protective effect of LINC01311 on an Aβ1–42-induced neuronal cell injury [61] (Figure 2).

### 2.3. LncRNAs Regulate Parkinson’s Disease by Modulating Autophagy

Parkinson’s Disease is the second most common chronic NDD that mainly afflicts people over 65 years of age [115]. Accumulating evidence indicates that PD pathogenesis involves a variety of cellular physiological processes, including oxidative stress, inflammatory responses, autophagy, mitochondrial dysfunction, ubiquitin–proteasome system dysfunction, apoptosis and various other processes [123,124,125]. The main characteristic of PD is the degenerative death of dopamine (DA)-producing neurons within the substantia nigra pars compacta (SNpc). The extensive loss of dopaminergic neurons reduces the levels of dopamine in the brain and leads to motor dysfunction. Multiple lines of evidence demonstrate that both genetic predisposition and external risk factors (aging, genetic susceptibility, environmental exposures) play crucial roles in the pathogenesis of PD. Mutations in the genes encoding for alpha-synuclein, LRRK2, PINK1 and PARK2 are associated with familial PD. The alpha-synuclein is a presynaptic neuronal protein; the abnormal expression and accumulation of alpha-synuclein lead to the formation of Lewy bodies (LBs) and Lewy nerve protrusions (LNs). These abnormal structures tend to accumulate in neurons within the brain, leading to neuronal cell death [126,127,128].

Results of recent studies have shown that autophagy is a promising therapeutic target for PD; lncRNAs regulate the pathogenesis of PD through modulating the autophagy signaling pathway. Several lncRNAs, such as NEAT1, HOTAIR (the lncRNA HOX transcript antisense RNA) and BDNF-AS (brain-derived neurotrophic factor-antisense), aggravate PD progression by activating autophagy. NEAT1 expression was significantly increased by 1-methyl-4-phenyl-1,2,3,6-tetrahydropyridine (MPTP) in both cell and mouse models of PD; NEAT1 promoted MPTP-induced autophagy by stabilizing PINK1 protein, and the knockdown of NEAT1 suppressed autophagy and alleviated dopaminergic neuronal injury [129]. NEAT1 was observed to directly target miR-374c-5p expression; silencing NEAT1 upregulated miR-374c-5p, suppressed autophagy and apoptosis and increased the ratio of tyrosine hydroxylase (TH^+^) neurons in MPTP-indued PD mice [130]. Similarly, Li et al. found that NEAT1 expression level was positively correlated with *N*-methyl-4-phenylpyridinium (MPP^+^) concentration, and interfering with NEAT1 dramatically repressed autophagy and apoptosis in PD mice via elevating the expression of miR-107-5p [131]. These findings indicate that NEAT1 plays a very important role in the pathophysiological process of PD; it can be considered as a promising diagnostic and therapeutic target for PD treatment. Recent studies have shown that HOTAIR can aggravate PD progression. HOTAIR was significantly upregulated in the substantia nigra compact tissues of PD mice, as well as in an MPP^+^ -treated PD cell model. HOTAIR could bind to miR-221-3p to elevate the expression of the miR-221-3p target, NPTX2 (neuronal pentraxin 2), thus enhancing autophagy of dopaminergic neurons both in vitro and in vivo [101]. Likewise, Zhao et al. found that HOTAIR promoted MPP^+^ -induced neuronal injury by sponging miR-874-5p in SK-N-SH cells. MiR-874-5p targeted the expression of ATG10, a key factor involved in autophagosome formation [102]. LncRNA BDNF-AS has been shown to be dysregulated in Parkinson’s disease; a recent study by Fan et al. reported that BDNF-AS was also upregulated in MPTP-induced PD mice, dopamine neurons and an MPP^+^ -induced SH-SY5Y cell model. Knockdown BDNF-AS significantly increased TH^+^ neurons, suppressed autophagy and increased cell viability by regulating the expression of miR-125-5p [103].

On the contrary, lncRNAs also promote Parkinson’s disease by inhibiting autophagy. For example, increased SNHG1 (small nucleolar RNA host gene 1) expression was observed in postmortem brain specimens of PD patients and PD cellular and animal models. Silencing SNHG1 promoted autophagy and prevented MPP^+^ -induced neuronal cell death [104,132]. SNHG1 could bind to the miR-221/222 cluster to trigger the expression of p27 and mTOR, thus activating mTOR signaling and inhibiting autophagy [104].

**Figure 3:**
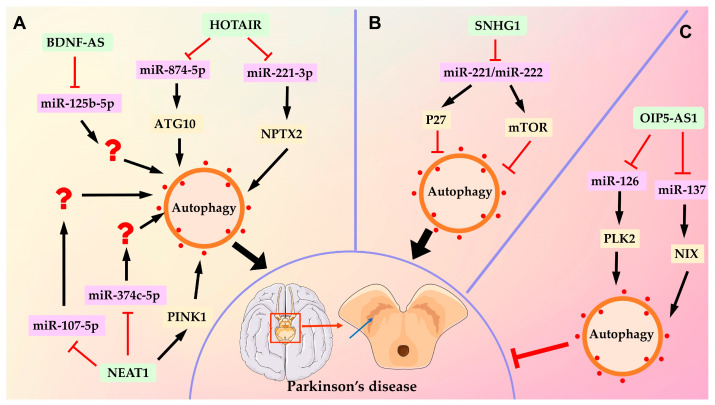
LncRNAs contribute to the pathogenesis of PD by modulating autophagy activity. Three different forms of functional cross-regulation between lncRNAs and autophagy are shown in PD: (**A**) LncRNA HOTAIR, BDNF-AS and NEAT1 promote PD progression by activating autophagy. NEAT1 can sponge miR-107-5p and miR-374c-5p or directly binds to PINK1 to activate autophagy. HOTAIR acts as a ceRNA to buffer the expression of miR-874-5p and miR-221-3p to upregulate ATG10- and NPTX2-mediated autophagy. BNDF-AS activates autophagy through miR-125b-5p. (**B**) LncRNA promotes PD pathogenesis by inhibiting autophagy. SNHG1 binds to miR-221/222 to upregulate P27 and mTOR expression, thus inactivating autophagy. (**C**) LncRNA OIP5-AS1 suppresses PD progression by activating autophagy. OIP5-AS1 promotes the expression of PLK2- and NIX-mediated autophagy by sponging miR-126 and miR-137, respectively. “?” means the target gene of miRNAs has not been identified in the paper.

LncRNA OIP5-AS1 has been shown to act as a major regulator of neurogenesis and plays a protective role in several neurological diseases [133]. OIP5-AS1 was downregulated in an MPP^+^ -treated PD cell model; the overexpression of OIP5-AS1 increased the expression of NIX through sponging miR-137, promoted mitochondrial autophagy and protected neuronal cells from degeneration [105]. OIP5-AS1 could also reduce the accumulation and toxicity of alpha-synuclein in MPP^+^ -treated SH-SY5Y cells by targeting miR-126 and upregulating the expression of PLK2 [106] (Figure 3). 

As mentioned above, lncRNAs carry out different functions and molecular mechanisms in NDDs by modulating autophagy. However, most studies have focused on the role of lncRNA regulation of autophagy in AD and PD; there is still much to do to explore the role of lncRNA-mediated autophagy regulation in the pathogenesis of other neurodegenerative disorders, such as HD and ALS. It is worth noting that the same lncRNA may produce opposite effects in different NDDs. For example, NEAT1 promotes the development of AD by inhibiting mitophagy, whereas in PD, NEAT1 aggravates PD by promoting cellular and mitochondrial autophagy. This paradox fully reflects the complexity and diversity of autophagy and lncRNAs in different types of NDDs.

## 3. Targeting Autophagy-Related lncRNAs as a Therapeutic Strategy for Neurodegenerative Diseases

NDDs will replace cancer as the second leading cause of human death after cardiovascular disease within 20 years [134]. Thus, it is very important and urgent to comprehensively investigate the pathogenic mechanisms of NDDs to improve the diagnosis and treatment of various neurological disorders. Drug development is challenged by the permeability of drugs in the blood–brain barrier (BBB) and the specificity of brain nerve cells [18]. Currently, there are very limited clinical applications for the treatment of NDDs, and the treatment is only symptomatic and has modest benefits. In AD, four chemicals (rivastigmine, galantamine, donepezil and memantine) and one amyloid-directed antibody have been approved by the FDA for the treatment of AD, but their effectiveness is not satisfying and varies from person to person [135]. In the last decade, a very large number of new AD drugs have been in clinical trials, but only one has been approved, with a failure rate of 99%. Most amyloid-based therapies have not shown any effect in clinical trials [136,137]. Of the 1761 clinical trials for PD, only 163 have been successfully completed, with a very low success rate as well [137]. This shows the huge obstacles facing the development of drugs that treat NDDs.

In addition to drugs, some new treatments such as gene therapy and stem cell therapy are being explored [138,139]. The absence of NGF (nerve growth factor) is associated with the pathogenesis of AD [140]. In clinical trials, NGF was delivered using adenovirus (AAV2)-mediated gene therapy. However, no significant differences were observed between patients in the treatment and placebo groups after one year [141]. There are two main therapeutic strategies in AAV2-mediated gene therapy for PD. The first is aimed at relieving clinical symptoms and targets either glutamic acid decarboxylase (GAD) or aromatic l-amino acid decarboxylase (AADC). The second approach focuses on restoring normal cellular function and transfers either glial derived neurotropic factor (GDNF) or the neurturin gene. However, clinical trials have proved that these methods have shortcomings of insufficient delivery or inaccurate positioning [142]. Therefore, the efficient, safe and specific delivery of gene products to the CNS remains a challenge.

Multiple lines of evidence demonstrate that autophagy is critical for the maintenance of homeostasis in neuronal cells; autophagy dysregulation is one of the main etiologies of NDDs, thus autophagy can be a very promising therapeutic direction for the treatment of neurodegenerative disorders [18,143]. Various types of small-molecule autophagy modulators, such as rapamycin, resveratrol, curcumin, berberine, etc., have been shown to display neuroprotective effects in experimental AD and PD models [144,145]. However, due to the lack of tissue and cell type specificity and substrate selectivity, no autophagy modulators have been successfully developed for clinical use. The identification of novel autophagy modulators boosting neuronal cell autophagy to specifically remove disease-related protein aggregates could be helpful in the therapeutic development for NDDs.

Given the ability of lncRNAs to regulate the development of NDDs through modulating cellular autophagy as described earlier, targeting autophagy-associated lncRNAs in neuronal cells is a potential therapeutic strategy for the treatment of NDDs. Currently, two major oligonucleotide-based strategies, antisense oligonucleotides (ASOs) and RNA interference (RNAi), have been proven successful in reducing the expression of upregulated lncRNAs in neuronal cells, indicating the huge therapeutic potential of RNA-based therapies for NDDs [146,147]. However, the main obstacle to the CNS is the BBB; oligonucleotides are unable to cross the BBB. Recent studies suggest that the combination of RNA-based therapies with liposomes is able to enhance BBB penetration [148]. Cell-derived exosomes are also considered as therapeutic vesicles to deliver RNAs to the CNS. The secondary structure of lncRNAs is probably another obstacle to their development as drugs, but the chemically modified analogs could be used to overcome the difficulty [149,150]. More importantly, the lack of conservation between lncRNA sequences in humans and experimental animal models will lead to further complications in the development of relevant therapies [151].

Currently, only a few lncRNAs were extensively studied to contribute to the pathogenesis of AD and PD by modulating the autophagy pathway (Table 2). Many aberrantly expressed lncRNAs that were detected by high-throughput sequencing need further experimental studies to understand their function and mechanisms in CNS disorders. With the development of new RNA-based therapeutic strategies, we believe that lncRNA could be a therapeutic treasure for NDDs.

## 4. Concluding Remarks

In this review, we summarize the functional cross-regulation between lncRNAs and autophagy in the context of neurodegenerative disorders, especially AD and PD. Brain tissues specifically express a large number of lncRNAs, so the investigation of the molecular mechanisms that connect lncRNAs and autophagy in NDDs will provide in-depth knowledge of brain physiology and therapy. The synergistic effects of lncRNA and autophagy may represent a novel and potentially effective therapeutic strategy in the treatment of NDDs, which still faces numerous challenges. Nevertheless, with a deep understanding of lncRNA biology and the technological development of nucleic-acid drugs, targeting autophagy-associated lncRNAs will have great potential for them to serve as effective diagnostic markers and therapeutic strategies.

## Figures and Tables

**Table 1:** Experimentally identified lncRNAs that regulate the pathogenesis of NDDs.

Alzheimer’s Disease		Parkinson’s Disease	
NEAT1 [26,27] MALAT1 [28,29] SNHG14 [30,31] HOTAIR [32,33] MAGI2-AS3 [34] XIST [35,36] GAS5 [37] BDNF-AS [38] BC200 [39] H19 [40] MEG3 [41] SNHG1 [42] ZBTB20-AS1 [43] WT1-AS [44] EBF3-AS [45]	00507 [46] TUG1 [47] SNHG19 [48] ANRIL [49] MIAT [50] SOX21-AS1 [51] RP11-59J16.2 [52] ATB [53] RPPH1 [54,55] LRP1-AS [56] 17A [57] MIR600HG [58] RMRP [59] BACE1-AS [60] LINC-01311 [61] SIX3OS1 [62]	NEAT1 [63,64] MALAT1 [65,66,67] MEG3 [68] DLX6-AS1 [69] H19 [70,71] TUG1 [72] RMST [73,74] JHDM1D-AS1 [75] NORAD [76,77] XIST [78] MIR17HG [79] FER1L4 [80] MIAT [81] BACE1-AS [82,83] ANRIL [84] SNHG14 [85] Linc-00667 [86]	SNHG15 [87] LINCRNA-P21 [88] UCA1 [89] LINC-00943 [90] HOTTIP [91] GAS5 [92,93] SNHG7 [94] SNHG12 [95] HAGLROS [96] PART1 [97] SOX2-OT [98] HOXA11-AS [99] HOXA-AS2 [100] HOTAIR [101,102] BDNF-AS [103] SNHG1 [104] OIP5-AS1 [105,106]

	
**Huntington’s Disease**		**Amyotrophic Lateral Sclerosis**	
NEAT1 [107,108]	DNM3OS [109] ABHD11OS [110]	NEAT1 [111]	ZEB1-AS1 [112]
MEG3 [107]			

**Table 2:** The role of autophagy-associated lncRNAs in the pathogenesis of neurodegenerative diseases.

Neurodegenerative Diseases	LncRNA	Role in Autophagy	Role in NDDs
**Alzheimer’s disease**	17A [57]	Inhibit	Promote
	NEAT1 [120]		
	MIR600HG [58]		
	SIX3OS1 [62]		
	RMRP [59]	Activate	Promote
	BACE1-AS [60]		
	LINC-01311 [61]	Inhibit	Suppress
**Parkinson’s disease**	NEAT1 [129,130,131]	Activate	Promote
	HOTAIR [101,102]		
	BDNF-AS [103]		
	SNHG1 [104]	Inhibit	Promote
	OIP5-AS1 [105,106]	Activate	Suppress
